# Food-Grade Expression of Manganese Peroxidases in Recombinant *Kluyveromyces lactis* and Degradation of Aflatoxin B_1_ Using Fermentation Supernatants

**DOI:** 10.3389/fmicb.2021.821230

**Published:** 2022-02-14

**Authors:** Yu Xia, Rui He, Ying Sun, Hangyu Zhou, Minjie Gao, Xiuyu Hu, Xiaobing Cui, Qianqian Cheng, Zhouping Wang

**Affiliations:** ^1^State Key Laboratory of Food Science and Technology, Jiangnan University, Wuxi, China; ^2^School of Food Science and Technology, Jiangnan University, Wuxi, China; ^3^State Key Laboratory of Biocatalysis and Enzyme Engineering, School of Life Sciences, Hubei University, Wuhan, China; ^4^Key Laboratory of Carbohydrate Chemistry and Biotechnology, Ministry of Education, Jiangnan University, Wuxi, China; ^5^China Biotech Fermentation Industry Association, Beijing, China; ^6^Anhui Heiwa Food-Jiangnan University Joint R & D Center, Anhui Heiwa Food Technology Co., Ltd., Bozhou, China

**Keywords:** mycotoxins, aflatoxin B_1_, degradation, *Kluyveromyces lactis*, food-grade

## Abstract

Aflatoxins are naturally occurring high-toxic secondary metabolites, which cause worldwide environmental contaminations and wastes of food and feed resources and severely threaten human health. Thus, the highly efficient methods and technologies for detoxification of aflatoxins are urgently needed in a long term. In this work, we report the construction of recombinant *Kluyveromyces lactis* strains GG799(pKLAC1-Phs*mnp*), GG799(pKLAC1-Plo*mnp*), GG799(pKLAC1-Phc*mnp*), and then the food-grade expression of the three manganese peroxidases in these strains, followed by the degradation of aflatoxin B_1_ (AFB_1_) using the fermentation supernatants. The expression of the manganese peroxidases was achieved in a food-grade manner since *Kluyveromyces lactis* is food-safe and suitable for application in food or feed industries. The inducible expression process of the optimal recombinant strain GG799(pKLAC1-Phc*mnp*) and the aflatoxin B_1_ degradation process were both optimized in detail. After optimization, the degradation ratio reached 75.71%, which was an increase of 49.86% compared to the unoptimized results. The degradation product was analyzed and determined to be AFB_1_-8,9-dihydrodiol. The recombinant strain GG799(pKLAC1-Phc*mnp*) supernatants degraded more than 90% of AFB_1_ in the peanut samples after twice treatments. The structural computational analysis for further mutagenesis of the enzyme PhcMnp was also conducted in this work. The food-grade recombinant yeast strain and the enzyme PhcMnp have potential to be applied in food or feed industries.

## Introduction

Aflatoxins are naturally occurring highly toxic secondary metabolites, mainly produced by several species of fungus genus *Aspergillus* such as *A. flavus*, *A. parasiticus*, and *A. nomius* (Ismail et al., 2018; von Hertwig et al., 2020). Together with other mycotoxins, aflatoxins had caused worldwide contaminations and wastes of food supplies and severely threatened human health (Pitt and Miller, 2017; Eskola et al., 2020). There are more than 20 types of structurally similar but different molecules of aflatoxins, in which aflatoxin B_1_ is the most prominent and dangerous type (Abrar et al., 2013; Loi et al., 2016). In 2012, AFB_1_ was classified as the Group 1 carcinogen (carcinogenic to humans) by the International Agency for Research on Cancer (IARC) (Haque et al., 2020). Further studies and conclusions by IARC and World Cancer Research Fund (WCRF) strongly supported the linkage of AFB_1_ to liver cancer and other types of cancer risks (Marques et al., 2019; Claeys et al., 2020). In China, the risks of exposure to aflatoxin B_1_ in foodstuffs or feeds still needed long-term supervision and control (Zhang et al., 2020). Thus, the highly efficient methods and techniques for detoxification of aflatoxin are urgently needed in a long term, to continuously minimize the loss of foodstuffs worldwide and reduce the harmful effects to human beings.

Currently, there are mainly three types of mycotoxin detoxification methods and techniques: physical, chemical, and biological. The physical methods are mainly solvent extraction and absorption, high-temperature degradation, and radiation processing (Di Gregorio et al., 2014; Ismail et al., 2018), and the chemical methods include structural degradation of toxins by some organic reagents such as aldehydes, oxidizing agents (Ismail et al., 2018), and ozone (Wang et al., 2016a,b). The biological methods usually utilize the alive microorganisms (Styriak et al., 2001; Wang et al., 2018), the expressed enzymes (Cao et al., 2011; Wang et al., 2019), and the microbial metabolites (Ghazvini et al., 2016) for structural degradation of toxins. In comparison with the biological methods, the physical and chemical methods have some defects such as the loss of nutrition and the residue of absorbent and chemical compounds, so that the safety and negative effects of using these methods should be continuously investigated (Peng et al., 2018). The biological methods degrade the mycotoxins with mild parameters and in more environment-friendly ways (Loi et al., 2017). However, the direct use of microorganisms still has limitations. For example, many foodstuffs are not suitable for inoculation and proliferation of microorganisms (Assaf et al., 2019), or else the nutrition contents and the texture characteristics might be changed (Peng et al., 2018). Therefore, utilization of extracellular enzymes and culture supernatants might be the best strategies for biological detoxification of mycotoxins (Cao et al., 2011; Feltrin et al., 2017; Zhao et al., 2020).

In this work, we report a new and safe microbial approach for degradation of aflatoxin B_1_ (AFB_1_) by the culture supernatants of three food-grade recombinant yeasts, which contained inducibly expressed manganese peroxidases. Manganese peroxidase was originally used in industrial and agricultural applications such as enzymatic bleaching of pulp, treatment of agricultural waste, and treatment of dye wastewater (Ha et al., 2001); in this work, we achieved food-grade expression of the enzymes in yeast and applied the enzyme for the degradation of AFB_1_ in foodstuff peanuts. The coding genes for manganese peroxidases PhsMnp, PloMnp, and PhcMnp, which originated from *Phanerochaete sordida*, *Pleurotus ostreatus*, and *Phanerochaete chrysosporium*, were respectively cloned to expression vector pKLAC1 and then expressed in the food-grade expression host *Kluyveromyces lactis* GG799, which is widely regarded as a food-safe yeast strain and is a suitable organism for the production of food enzymes (van den Dungen et al., 2021). The recombinant strain GG799(pKLAC1-Phc*mnp*) with the highest AFB_1_ degradation efficiency was further studied, and the AFB_1_ degradation parameters were optimized. Besides, the structure of the manganese peroxidase PhcMnp was homology-modeling analyzed for further mutagenesis of this enzyme.

## Materials and Methods

### Chemicals, Strains, and Culture Techniques

AFB_1_ was purchased from Pribolab (Qingdao, China), which was made into a 1.0-mg/ml stock solution with methanol or acetonitrile solvent and was preserved and refrigerated in the darkness at −20°C. DNA marker, restriction enzymes (*Bgl*II, *Sal*I, *Sac*II, etc.), T4 DNA ligase, and plasmid extraction kits were purchased from Thermo Fisher Scientific (Shanghai, China). Sorbitol and hemin were purchased from Aladdin (Shanghai, China). Fungus genomic DNA extraction kit and growth media components were purchased from Sangon Biotech (Shanghai, China). Methanol, acetonitrile, and formic acid were purchased from Tedia (Fairfield, OH, United States). All other reagents and chemicals were of analytical reagent grade. The genome DNA of *K. lactis* recombinants was extracted and purified by the fungus genomic DNA extraction kit. All the DNA sequencing works were done in Sangon Biotech (Shanghai, China).

For cultivation of *K. lactis* GG799 and the recombinant strains GG799(pKLAC1-Phs*mnp*), GG799(pKLAC1-Plo*mnp*), and GG799(pKLAC1-Phc*mnp*), the strains were grown in YEPD liquid (1.0% yeast extract, 2.0% peptone, 2.0% glucose, and pH 6.3) at 30°C and 200 rpm for 18 h. For the inducible expression of manganese peroxidases in the recombinant strains, the strains were cultivated in YEPG medium (1.0% yeast extract, 2.0% peptone, 2.0% galactose, 1.0 mmol/l MnSO_4_, 1.0 mmol/l hemin, pH 6.5) under aerobic conditions at 30°C and 200 rpm for 96 h.

### Construction of Recombinant Plasmids and Expression Strains

**FIGURE 1:**
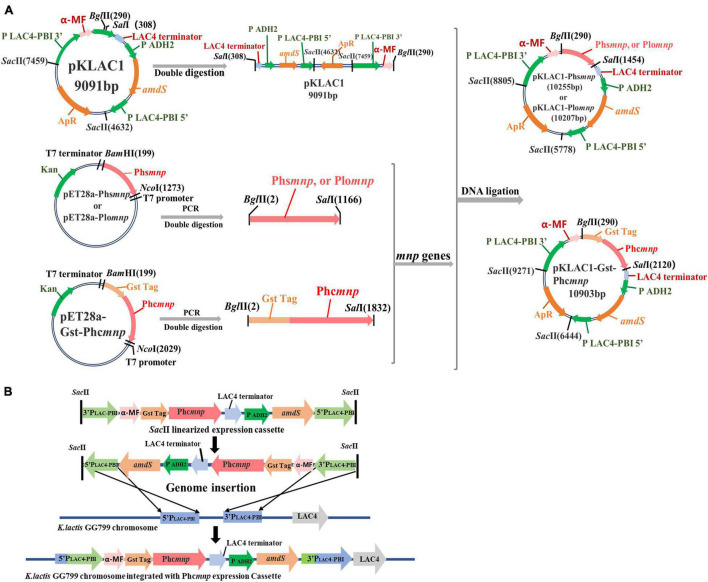
Flowchart for constructions of recombinant plasmids and strains. **(A)** Flowchart for constructions of recombinant plasmids. **(B)** The integration mechanism for construction of recombinants using *K. lactis* GG799(pKLAC1-Phc*mnp*) as an example.

Three manganese peroxidases known to be capable of degrading AFB_1_ in the original strains were selected. Two manganese peroxidase genes from *P. sordida* (Wang et al., 2011) and *P. ostreatus* (Yehia, 2014) were synthesized and named Phs*mnp* and Plo*mnp*, respectively, and the manganese peroxidase gene from *P. chrysosporium* (Gowda et al., 2007) was constructed and named Phc*mnp* by adding a glutathione-S-transferase (GST) tag to its 5′-end, for the GST tag can be co-expressed with proteins to increase the solubility of easily aggregated proteins (Tarazi et al., 2021). The three synthesized genes were inserted in the pKLAC1 vector to the *Bgl*II and *Sal*I sites and then linked by T4 DNA ligase to obtain the constructs (pKLAC1-Phs*mnp*, pKLAC1-Plo*mnp*, and pKLAC1-Phc*mnp*). These plasmids were transformed into the host strain *E. coli* DH5α for screening of positive clones by PCR verification (detailed PCR program shown in Supplementary Table 1), restriction digestion of the plasmids, and DNA sequencing. The verified recombinant plasmids were amplified in the *E. coli* hosts and then transformed into the yeast host *K. lactis* GG799, for the construction of recombinant yeast strains. Detailed construction procedures for recombinant plasmids and yeast strains are shown in Figure 1. The result recombinant yeast strains were named GG799(pKLAC1-Phs*mnp*), GG799(pKLAC1-Plo*mnp*), and GG799(pKLAC1-Phc*mnp*), respectively.

### Inducible Expression Methods of the Enzymes

The recombinant strains *K. lactis* GG799(pKLAC1-Phs*mnp*), GG799(pKLAC1-Plo*mnp*), and GG799(pKLAC1-Phc*mnp*) were cultivated in YEPD liquid. When OD_600_ reached 1.0, 1% of the cultures were transferred into the YEPG liquid for the inducible expression of the manganese peroxidases, according to the methods described above. The supernatants of these cultures were collected by centrifugation and concentrated by 10-kDa ultrafiltration for subsequent experiments.

### Degradation System of AFB_1_ by the Enzymes

The AFB_1_ degradation process was conducted with the supernatants obtained from the recombinant strains GG799(pKLAC1-Phs*mnp*), GG799(pKLAC1-Plo*mnp*), and GG799(pKLAC1-Phc*mnp*). The total volume of the reaction system was 1.0 ml, containing 50.0 mmol/l malonic acid buffer (pH 4.5), 2.0 μg/ml AFB_1_, 1.0 mmol/l MnSO_4_, 2.0 mmol/l glucose, 1.0 U/ml glucose oxidase, and 1.0 mg/ml culture supernatant protein, while the protein was replaced with 50.0 mmol/l malonic acid buffer (pH 4.5) in blank control. The solution containing all the samples needed was incubated at 40°C for 40 h, followed by enzyme inactivation in a water bath at 80°C for 10 min. The samples were filtered through the 0.22-μm membranes for removal of the impurities and then subjected to UPLC-TQD analysis.

### Quantitative Assay of AFB_1_

The UPLC-TQD assay was used for qualitative and quantitative analyses of the concentration of AFB_1_ in samples. The specific operating parameters were as follows, UPLC chromatographic conditions: column: C18; flow rate: 0.30 ml/min; column temperature: 40°C; mobile phases: H_2_O (buffer A) and acetonitrile (buffer B). MS (Mass) spectrometry conditions: ion source: electrospray ion source; MS spectrometry scanning mode: multiple reaction monitoring mode (MRM); cone hole voltage: 3.0 kV; heating gas temperature: 500°C; ion source temperature: 150°C; desolvation gas: 800 l/h.

The AFB_1_ standard samples were prepared in acetonitrile with the concentration gradients (100.0, 200.0, 500.0, 1,000.0, 2,000.0, and 5,000.0 ng/ml) to establish the standard curve for AFB_1_ concentration calculation.

### Optimization of Inducible Expression Conditions for GG799(pKLAC1-Phc*mnp*)

The recombinant strain *K. lactis* GG799(pKLAC1-Phc*mnp*) was subjected to different induction conditions (temperature: 15–35°C, time: 24–120 h, rotation speed: 0–300 rpm, hemin concentration: 0.1–5.0 mmol/l, initial pH of induction medium: 3.0–9.0, MnSO_4_ concentration: 0.1–5.0 mmol/l, galactose concentration: 5.0–80.0 g/l). The enzyme was expressed and secreted to the supernatants as reported (Gnanamani et al., 2006). The expression levels of enzyme PhcMnp were optimized with these parameters univariately and characterized by the degradation ratio of AFB_1_.

On the basis of the single-factor test, seven factors (temperature, time, rotation speed, hemin concentration, initial pH of induction medium, MnSO_4_ concentration, and galactose concentration) with different levels were designed for the orthogonal test. The test factors and levels are shown in Supplementary Table 2.

### Optimization of Degradation Parameters for AFB_1_ Degradation by Supernatants of GG799(pKLAC1-Phc*mnp*)

Under the optimal induced expression conditions, the degradation reaction conditions for AFB_1_ by the supernatants of recombinant strain GG799(pKLAC1-Phc*mnp*) were further optimized by setting the concentration gradient, according to the methods reported previously (Wang et al., 2011). For the optimization of single factors by testing the degradation ratio under different reaction conditions, reaction time (4–48 h), temperature (20–60°C), pH (3.0–5.5), protein concentration (0.4–8.0 g/l), MnSO_4_ concentration (0.2–10.0 mmol/l), glucose concentration (1.0–3.5 mmol/l), and glucose oxidase concentration (0.2–5.0 U/ml) were investigated.

Seven factors (time, temperature, pH, protein concentration, MnSO_4_ concentration, glucose concentration, and glucose oxidase concentration) and levels of the orthogonal test were also designed (Supplementary Table 3).

### Degradation Methods of AFB_1_ in Peanuts by the Enzymes

According to the methods reported previously (Yang et al., 2020), the peanut samples were shelled, the impurities and dust of which were removed. The samples were ground into powders by homogeneity, with the particle size less than 2.0 mm. The powder samples (5.0 g) were added with 10.0 ml water, stirred thoroughly, followed by sterilization in an autoclave.

The AFB_1_ standards were added to the peanut samples at different concentrations (50.0, 500.0, and 2,000.0 μg/kg), and the samples were stirred thoroughly. For AFB_1_ degradation tests, the samples were mixed with 1.2 mmol/l MnSO_4_, 2.5 mmol/l glucose, 1.5 U/ml glucose oxidase, and the supernatants (containing proteins concentration: 3.0 g/l) for enzymatic degradation reactions. The final volume of the reaction system was 35.0 ml. The reaction was carried out at 40°C, pH of 4.5, for 40 h. All the samples were analyzed by HPLC-MS spectrum (UPLC-TQD) assay to detect the residual concentration of AFB_1_ in the samples.

## Results

### Construction of Recombinant

Strains *Kluyveromyces lactis* GG799(pKLAC1-Phs*mnp*), GG799(pKLAC1-Plo*mnp*), and GG799(pKLAC1-Phc*mnp*).

Three recombinant plasmids pKLAC1-Phs*mnp*, pKLAC1-Plo*mnp*, and pKLAC1-Phc*mnp* were constructed in this work according to the methods described above. The flowchart for the construction is shown in Figure 1A. The vector pKLAC1 and genes Phs*mnp*, Plo*mnp*, and Phc*mnp* were 9,091, 1,164, 1,116, and 1,824 bp (with a GST tag fused on the 5′-terminal), respectively. Plasmid verification results using agarose gel electrophoresis are shown in Supplementary Figure 1, and the plasmids with correct digestion results were subjected to DNA sequencing verification.

The recombinant plasmids were transferred *K. lactis* GG799 host, and the transformants were selected and verified by PCR amplification, as shown in Supplementary Figure 2, which indicated successful construction of the recombinant strains of *K. lactis* GG799(pKLAC1-Phs*mnp*), GG799(pKLAC1-Plo*mnp*), and GG799(pKLAC1-Phc*mnp*).

### Inducible Expression of Enzymes and Degradation of AFB_1_ by the Culture Supernatants of the Recombinant Strains

To express inducibly for manganese peroxidases, the recombinant strains GG799(pKLAC1-Phs*mnp*), GG799(pKLAC1-Plo*mnp*), and GG799(pKLAC1-Phc*mnp*) were firstly cultivated in YEPD liquid for accumulation of the biomass and then induced in YEPG liquid.

**FIGURE 2:**
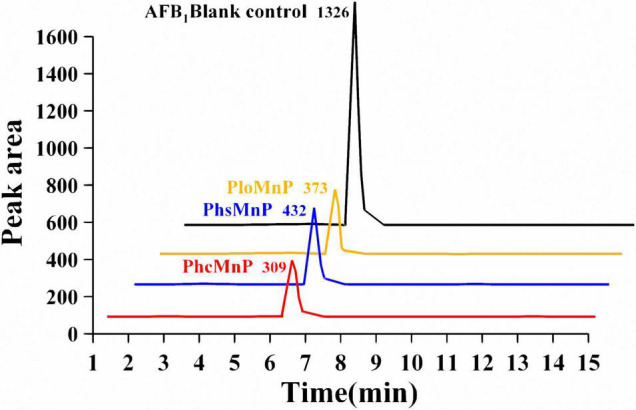
Comparison of liquid chromatogram of AFB_1_ degradation reaction by GG799(pKLAC1-Plo*mnp*), GG799(pKLAC1-Phs*mnp*), and GG799(pKLAC1-Phc*mnp*). The addition amount of three enzymes in the system was 1.0 mg/ml. The black peak was AFB_1_ blank control; the orange peak was the residual of AFB_1_ degraded by enzyme PloMnp; the blue peak was the residual of AFB_1_ degraded by PhsMnp; and the red peak was the residual of AFB_1_ degraded by PhcMnp. All the peaks in this 3D visualization figure were appeared at the retention time of 6.3 min.

The activities of manganese peroxidases were characterized by AFB_1_ degradation ratios. The supernatants of the recombinant strains were used for AFB_1_ degradation, and the remaining contents of AFB_1_ were analyzed by HPLC-TQD. A comparison of the liquid chromatogram of AFB_1_ degradation results by the three recombinants’ supernatants is shown in Figure 2. It can be seen that the peak area of degradation residue by PhcMnp was the smallest in the three samples, which indicated that the enzyme PhcMnp has the best enzyme activity. The degradation ratios of AFB_1_ by the three recombinants’ supernatants are shown in Table 1. As shown in this table, the supernatant of recombinant strain GG799(pKLAC1-Phc*mnp*) had the highest degradation ratio for AFB_1_ (50.52 ± 3.69%), which was significantly higher than those from the recombinant strains GG799(pKLAC1-Phs*mnp*) (35.55 ± 3.30%) and GG799(pKLAC1-Plo*mnp*) (40.02 ± 1.77%) (*p* < 0.05).

**TABLE 1:** — Degradation of AFB_1_ by manganese peroxidase recombinants’ culture supernatants.

Manganese peroxidase recombinants from different sources	GG799(pKLAC1-Phs*mnp*)	GG799(pKLAC1-Plo*mnp*)	GG799(pKLAC1-Phc*mnp*)
Degradation ratio (%)	35.55 ± 3.30^b^	40.02 ± 1.77^b^	50.52 ± 3.69^a^

*Three parallel reactions were done in each group, and different lowercase letters (a, b) indicate significant differences in the degradation rate of AFB_1_ between samples.*

### Optimization of Inducible Expression for GG799(pKLAC1-Phc*mnp*) and the Degradation Efficiency of AFB_1_

The influence of factors such as induction temperature, time, and rotation speed on degradation ratios of AFB_1_ was designed to analyze the expression level for the enzyme PhcMnp. As shown in Supplementary Figures 3, 4, the degradation ratios observed by the recombinants’ supernatants on AFB_1_ under the conditions of different induction factors had similar trends. With the increase in induction temperature, time, rotation speed, medium pH (Supplementary Figures 3A–D), and the increased concentrations of hemin, MnSO_4_, and galactose (Supplementary Figures 4A–C), the degradation ratios of AFB_1_ showed a trend of increasing at first and then decreasing.

In Supplementary Figure 4A, a high concentration of hemin inhibited the enzyme activity and degradation, causing low degradation efficiency of AFB_1_, which was similar to the previous results (Jiang et al., 2008).

The results in Supplementary Figure 4B indicated that with the increase in MnSO_4_ concentration in the medium, the degradation effect on AFB_1_ increased at first and then decreased, and the highest degradation ratio of AFB_1_ reached 58.32 ± 2.07% at the MnSO_4_ concentration of 1.0 mmol/l. Meanwhile, the expression level of the enzyme GG799(pKLAC1-Phc*mnp*) was also influenced by the concentration of the inducer galactose (Xia et al., 2021). In Supplementary Figure 4C, the highest degradation ratio of AFB_1_ reached 53.48 ± 1.63% at the MnSO_4_ concentration of 60 g/l.

From the above results, the optimal conditions for the inducible expression of the AFB_1_ degradation enzyme were as follows: YEPG medium containing 60.0 g/l galactose, 1.0 mmol/l MnSO_4_, 1.0 mmol/l hemin, and an initial medium pH of 6.0, with induction at 30°C and 200 rpm for 96 h.

To further explore the optimal combination of the induction expression conditions of GG799(pKLAC1-Phc*mnp*), factors like induction temperature, induction time, rotation speed, hemin concentration, initial pH of the medium, MnSO_4_ concentration, and galactose concentration were designed in an orthogonal experiment, and the optimization results were characterized by the degradation ratio of AFB_1_. The results are shown in Supplementary Table 4. The optimum induction parameter combination was A_2_D_2_B_2_F_2_C_2_E_3_G_1_. Under this condition, the highest degradation ratio of AFB_1_ by the recombinant’s supernatant was 67.40 ± 0.74%, which was increased by 15.6% compared to the optimal result of the single-factor optimization test.

### Optimization of Reaction Parameters for AFB_1_ Degradation and Determination of the Results

**FIGURE 3:**
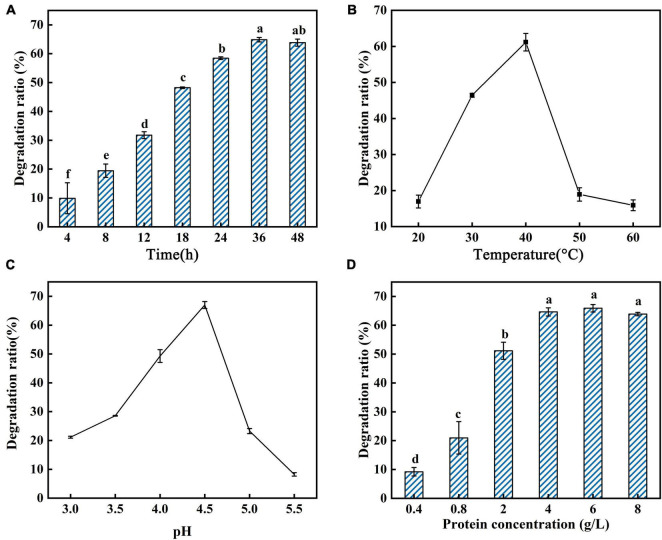
Degradation ratio of AFB_1_ by PhcMnp under different reaction parameters. **(A)** The effects of time on the degradation ratio of AFB_1_ by PhcMnp. **(B)** The effects of temperature on the degradation ratio of AFB_1_ by PhcMnp. **(C)** The effects of pH on the degradation ratio of AFB_1_ by PhcMnp. **(D)** The effects of protein concentrations on the degradation ratio of AFB_1_ by PhcMnp.

**FIGURE 4:**
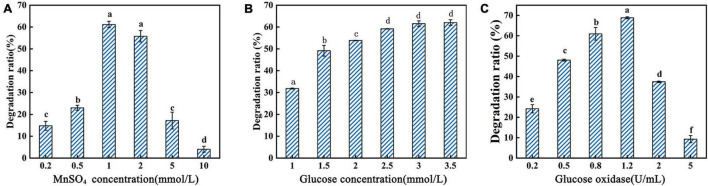
Degradation ratio of AFB_1_ by PhcMnp under different concentrations of reaction additives. **(A)** The effects of MnSO_4_ concentrations on the degradation ratio of AFB_1_. **(B)** The effects of glucose concentrations on the degradation ratio of AFB_1_. **(C)** The effects of glucose oxidase concentrations on the degradation ratio of AFB_1_.

In this study, the reaction conditions for AFB_1_ degradation by supernatant of GG799(pKLAC1-Phc*mnp*) were optimized with the following factors: reaction time, reaction temperature, pH, protein concentration, MnSO_4_ concentration, glucose addition, and glucose oxidase addition, and the results are shown in Figures 3, 4. As shown in Figure 3, the trends of the degradation ratio of AFB_1_ under different reaction parameters were approximately similar. With the increase in induction time, temperature, pH, and protein concentration (Figures 3A–D), the degradation ratio of AFB_1_ by PhcMnp showed a trend of increasing at first and then decreasing.

As shown in Figure 4A, a high concentration of MnSO_4_ in the medium inhibited the degradation ratio of AFB_1_. The highest degradation ratio of AFB_1_ by the recombinant’s supernatant reached 61.16 ± 1.09% at the MnSO_4_ concentration of 1.0 mmol/l; however, when the MnSO_4_ concentration was 10.0 mmol/l, the degradation ratios of AFB_1_ were less than 10%. The degradation of AFB_1_ by the enzyme requires the participation of H_2_O_2_, but due to the instability of H_2_O_2_ and the reversible inactivation of manganese peroxidase under the condition of a high concentration of H_2_O_2_ (Wariishi et al., 1988), we chose to add glucose and glucose oxidase to the reaction system to release H_2_O_2_ slowly and steadily in this study, as shown in Figures 4B,C. In Figure 4B, the degradation ratio of AFB_1_ increased with the addition of glucose and then decreased when the glucose addition was greater than 2.5 mmol/l, probably because the H_2_O_2_ produced by the catalysis of 1.5 U/ml glucose oxidase at the beginning of the addition was not sufficient for the degradation of 2.0 μg/ml AFB_1_. Conversely, when the glucose addition reached 2.5 mmol/l, the amount of H_2_O_2_ produced by the catalysis of 1.5 U/ml glucose oxidase was saturated.

From the above results, the optimal reaction parameters for AFB_1_ degradation by GG799(pKLAC1-Phc*mnp*) supernatants were as follows: degradation system containing 6.0 g/l protein, 1.0 mmol/l MnSO_4_, 3.5 mmol/l glucose, and 1.2 U/ml glucose oxidase, pH of 4.5, with induction at 40°C for 36 h.

To further explore the optimal combination of the induction reaction parameters of GG799(pKLAC1-Phc*mnp*), factors were designed in an orthogonal experiment, and the optimization results were characterized by the degradation ratio of AFB_1_. The orthogonal experimental results are shown in Supplementary Table 5. The optimum induction proposal was C_2_B_2_D_3_F_2_A_3_E_1_G_3_. The highest degradation ratio of AFB_1_ by the recombinant’s supernatant was 75.71 ± 1.21% under this optimum induction proposal, which was increased by 9.1% compared to the optimal result of the single-factor optimization test.

### Analysis of AFB_1_ Degradation Products by GG799(pKLAC1-Phc*mnp*) Supernatants

**FIGURE 5:**
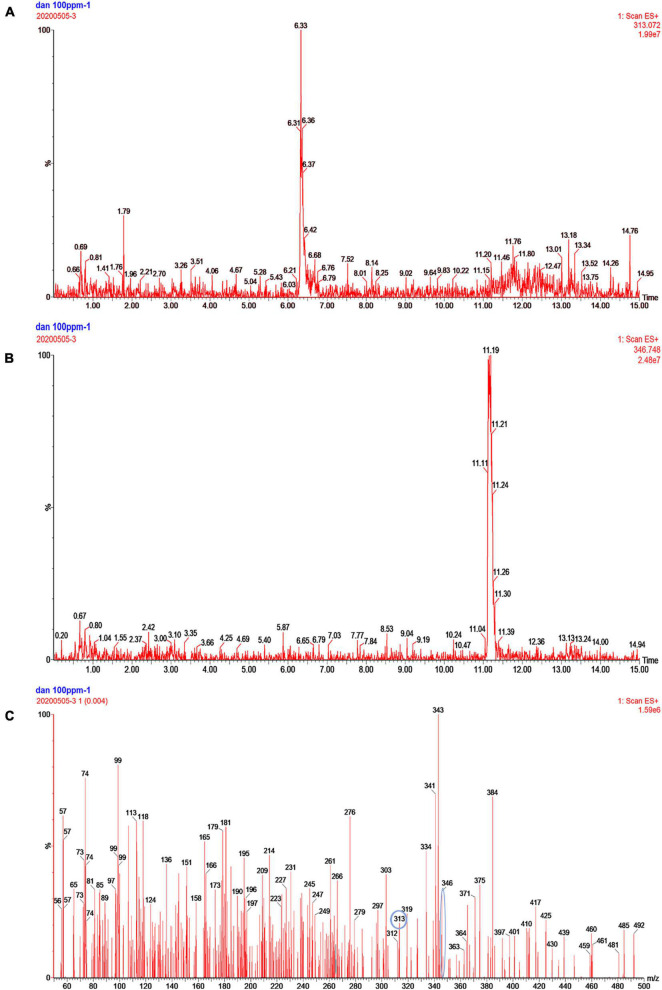
Analysis of the degradation products of AFB_1_ by GG799(pKLAC1-Phc*mnp*) fermentation broth. **(A)** Chromatogram of AFB_1_ metabolites degraded by fermentation broth of GG799(pKLAC1-Phc*mnp*). The chromatogram peak at retention time of 6.3 min was AFB_1_. **(B)** Chromatogram of the AFB_1_-8,9-dihydrodiol standard. The chromatogram peak near the retention time of 11.19 min was AFB_1_-8,9-dihydrodiol standard. **(C)** Mass spectrum of AFB_1_ degradation product by fermentation supernatants of GG799(pKLAC1-Phc*mnp*). [M + H]^+^ ions with a mass-to-charge ratio (m/z) of 313 (circled) were AFB_1_, and those of slightly larger than 346 (circled) were presumed to be AFB_1_-8,9-dihydrodiol.

**FIGURE 6:**
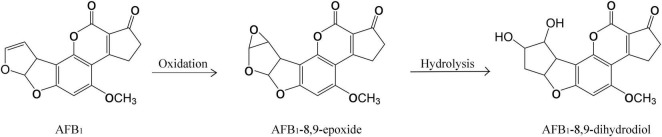
Schematic for degradation of AFB_1_ by manganese peroxidases.

The AFB_1_ degradation products treated with fermentation broth of GG799(pKLAC1-Phc*mnp*) were identified by the mass spectrum, and the results are shown in Figure 5. The chromatogram of AFB_1_-degraded results by GG799(pKLAC1-Phc*mnp*) is shown in Figure 5A, in which the AFB_1_ retention time was found at 6.33 min. In addition, a large number of chromatogram peaks were found near the retention time of 11.15 min, which was considered to be the retention time of AFB_1_-8,9-dihydrodiol. This compound was regarded as the major degradation product of AFB_1_ treated with GG799(pKLAC1-Phc*mnp*) supernatants. To confirm this, the AFB_1_-8,9-dihydrodiol standards were subjected to UPLC-TQD analysis, and the retention time of this compound was found near 11.15 min (Figure 5B), whose result was consistent with that shown in Figure 5A. Theoretically, the molecular weights of AFB_1_ and AFB_1_-8,9-dihydrodiol are 312.27 and 346.288, respectively. The mass spectra of the samples of AFB_1_ treated with the supernatant of recombinant GG799 (pKLAC1-Phc*mnp*) in MRM scan mode are shown in Figure 5C, the [M + H]^+^ ions with a mass-to-charge ratio (m/z) of 313 were AFB_1_, and those slightly larger than 346 were presumed to be AFB_1_-8,9-dihydrodiol, which was consistent with the conclusion of Wang et al. (2011), who found that AFB_1_-8,9-dihydrodiol produced by the degradation of AFB_1_ was much less toxic than AFB_1_. The schematic for the degradation of AFB_1_ by manganese peroxidases is shown in Figure 6.

### Degradation Efficiency of AFB_1_ in Peanuts

To investigate the degradation ratio of GG799(pKLAC1-Phc*mnp*) supernatant in the actual samples, degradation experiments were conducted using peanut samples added with AFB_1_. The degradation results are shown in Table 2. The recombinant strain GG799(pKLAC1-Phc*mnp*) supernatant degraded more than 90% of AFB_1_ in the peanut samples after twice treatments, with no significant differences (*p* > 0.05). When the AFB_1_ concentration was 50.0 μg/kg, the standard deviation was larger due to the low AFB_1_ content in the contaminated peanut samples. Besides, the larger the AFB_1_ concentration used, the smaller the standard deviation was found. These consistent data indicated that the GG799(pKLAC1-Phc*mnp*) culture supernatants were suitable for practical usage.

**TABLE 2:** — Degradation results of AFB_1_ in peanut samples by GG799(pKLAC1-Phc*mnp*) culture supernatants.

Experiment number	Concentration of AFB_1_ added in samples (μ g/kg)	Concentration of AFB_1_ detected in samples (μ g/kg)	Concentration of AFB_1_ detected after once treatment (μ g/kg)	Degradation ratio after once treatment (%)	Concentration of AFB_1_ detected after twice treatments (μ g/kg)	Degradation ratio after twice treatments (%)
1	50.0	43.77	7.59	82.66 ± 13.17	–	*82.66 ± 13.17
2	500.0	417.96	98.31	76.48 ± 1.99	27.67	93.38 ± 0.93
3	2000.0	1766.39	368.26	79.15 ± 0.07	146.39	91.71 ± 3.99

Three parallel reactions were done in each group, and the standard deviations were listed. *Degradation ratio after the second treatment in experiment number 1 followed the first degradation ratio.

### Mutagenesis Site Prediction of the Manganese Peroxidase PhcMnp

**FIGURE 7:**
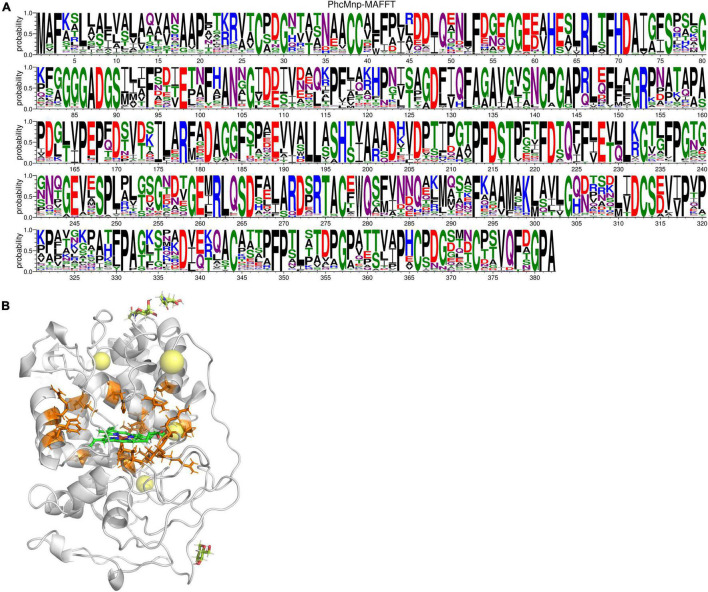
Amino acid sequence and structure analysis of PhcMnp. **(A)** Amino acid evolutionary conservation distribution of PhcMnp. The size of symbols within the stack indicates the relative frequency of each amino acid at that position. **(B)** Homology model of PhcMnp. The model structure of PhcMnp is constructed with the crystal structure of MnP-Cd*^II^* (PDB ID: 1YYG) as a template. The predicted single mutant sites are shown in orange. The heme ring is shown in green.

Single variants of PhcMnp were predicted based on the consensus approach, folding free energy calculations, and some structural considerations. On the one hand, amino acid evolutionary conservation distribution (Figure 7A) was obtained as follows: the amino acid sequence of PhcMnp was used to identify the homologous sequences (>40% sequence identity) from the NCBI database. After removing the redundant sequences through the CD-HIT program (Huang et al., 2010) with a 90% identity threshold, the 365 resulting sequences were aligned using MAFFT software (Katoh and Standley, 2013). The amino acid distributions at each position corresponding to that of PhcMnp were analyzed by WebLogo.^1^ On the other hand, we constructed a structural model of PhcMnp (Figure 7B) based on the crystal structures of its homolog enzyme MnP-Cd*^II^* (PDB ID: 1YYG, 1.6 Å, 84% identity to PhcMnp). The software YASARA was used to perform the homology modeling. Alanine-scanning mutagenesis (Chauhan, 2017) was carried out on the homology model of PhcMnp using the Calculate Mutation Energy (Stability) protocol of Discovery Studio 2020 Client. Mutation energy prediction showed that six variants (70H, 110S, 174K, 178R, 192S, and 243T) had a much lower energy than others. These sites were then selected for full mutation scanning. Considering the conservation and energy, it was reasoned that the seven single variants (H70D, S110A, K174S, R178E, S192A, T243Q, and T243P) may have potentially enhanced activity. Besides, based on the criteria of non-mutation of highly conservative amino acids, low mutation energy, and being close to heme (<5 Å), it was reasoned that the 13 single variants (E59A, I65L, N105G, N105A, N105L, L138V, F179M, V199I, R201A, K204V, K204E, V205I, and L300M) may also have potentially enhanced activity.

## Discussion

Manganese peroxidase is of increasing interest due to its potential for industrial applications, which was originally used in the treatment of agricultural waste and the lignin degradation (Ha et al., 2001). However, we used manganese peroxidase to achieve food-grade yeast expression in *K. lactis* GG799 for AFB_1_ degradation in this work.

The expression level for the enzyme PhcMnp is mainly influenced by factors such as induction temperature, time, and rotation speed; meanwhile in this work, the inducible expression for the enzyme PhcMnp was also regulated by the concentration of hemin and Mn^2+^. Hemin is a non-covalently bound prosthetic group in MnP, which is crucial to activating soluble MnPs (Wang et al., 2019). Also, manganese peroxidase consists of a ferric heme group and Mn^2+^ as its active center, and previous reports (Whitwam and Tien, 1996; Gettemy et al., 1998) showed that the manganese peroxidase genes are regulated by Mn^2+^ at the transcriptional level, and a certain amount of Mn^2+^ may promote the synthesis of manganese peroxidase.

As for the degradation of AFB_1_, the catalytic reaction of manganese peroxidase (MnP) requires the participation of H_2_O_2_ and Mn^2+^. The Fe^3+^-containing MnP is oxidized by H_2_O_2_ to produce complex I (Fe^4+^–oxygen–porphyrin radical complex), and then complex I is reduced by Mn^2+^ to produce complex II (Fe^4+^–oxygen–porphyrin complex) by a single electron. Finally, complex II is reconverted to MnP and Mn^3+^ by using Mn^2+^ as an electron donor (Hofrichter, 2002). The product Mn^3+^ can form chelates with some organic acids (e.g., oxalic acid, malonic acid, and malic acid) to oxidize complex organics to achieve the degradation of AFB_1_ (Wang et al., 2019). Therefore, the addition of Mn^2+^ in the reaction system is necessary, but some reports (Xia et al., 2021) showed that the presence of excessive Mn^2+^ may also inhibit the enzymatic activity of manganese peroxidase.

The degradation product of AFB_1_ was analyzed and determined to be AFB_1_-8,9-dihydrodiol in this work. The main pathway of degradation of AFB_1_ was through the free radicals generated by the interaction of oxidized Mn^3+^ with the dicarboxylic acid malonate (Wang et al., 2019), which increased the redox potential for oxidization of AFB_1_. In the degradation process, the intermediate AFB_1_-8,9-epoxide was produced before AFB_1_-8, 9-dihydrodiol, and it would be rapidly hydrolyzed to AFB_1_-8,9-dihydrodiol. The degradation product AFB_1_-8,9-dihydrodiol was greatly less toxic than AFB_1_, since it has no binding sites for DNA, lipids, and other macromolecules, thus achieving the purpose of detoxification of AFB_1_ (Wang et al., 2019).

As for the degradation ratio of AFB_1_, the recombinant laccase expressed in *Aspergillus niger* (118.0 U/l) achieved 55% of the degradation ratio for AFB_1_ (Alberts et al., 2009), and manganese peroxidase produced by *Phanerochaete sordida* YK-624 achieved 86% of the degradation ratio for AFB_1_ (Wang et al., 2011). Doyle and Marth (1979) found that lactoperoxidase in *Aspergillus parasiticus* could degrade AFB_1_ with up to 30.4% degradation ratio. However, the degradation ratio of AFB_1_ in the peanuts by the manganese peroxidase in this work reached more than 90%, which was much higher than those results by the enzymes produced in wild-type strains.

In conclusion, the manganese peroxidases used in this study were all derived from food-safe microorganisms (*Phanerochaete sordida*, *Pleurotus ostreatus*, and *Phanerochaete chrysosporium*) and the recombinant enzyme PhcMnp performed well in the degradation of AFB_1_. The food-grade expression system including the host *K. lactis* GG799 and the vector pKLAC1 was used in this work, in which the yeast *K. lactis* has been widely used in the food, feed, and pharmaceutical industries without environmental risks (Spohner et al., 2016). Therefore, this study provides a basis for subsequent large-scale fermentation of food-grade recombinant enzymes for degradation of mycotoxins in foodstuffs or feedstuffs. Furthermore, results showed that the enzyme PhcMnp has potential for practical application in the food industry. Besides, mutagenesis site prediction of the manganese peroxidase PhcMnp provided the theoretical basis for the following experiments in improvement or property modification of the enzyme.

## Data Availability Statement

The datasets presented in this study can be found in online repositories. The names of the repository/repositories and accession number(s) can be found in the article/Supplementary Material.

## Author Contributions

YX conceived the research strategies, designed the experiments, gave important guidance to the experiments, and assisted in completing the final manuscript. RH designed the experiments, conducted the experiments, and prepared the drafts of the manuscript. YS conducted the experiments and analyzed the data. HZ conducted the protein structure analysis. MG participated in the mycotoxin degradation experiments in practical examples. XH, XC, and QC provided partial materials, investigation data, and some experimental technique supports. ZW provided guidance and partial financial supports for the experiments. All authors read and approved the final manuscript.

## Conflict of Interest

XC is employed by Anhui Heiwa Food Technology Co., Ltd. The remaining authors declare that the research was conducted in the absence of any commercial or financial relationships that could be construed as a potential conflict of interest.

## Publisher’s Note

All claims expressed in this article are solely those of the authors and do not necessarily represent those of their affiliated organizations, or those of the publisher, the editors and the reviewers. Any product that may be evaluated in this article, or claim that may be made by its manufacturer, is not guaranteed or endorsed by the publisher.

## Footnotes

1
https://weblogo.berkeley.edu/logo.cgi
