# Supplementary material for: Food-Grade Expression of Manganese Peroxidases in Recombinant Kluyveromyces lactis and Degradation of Aflatoxin B1 Using Fermentation Supernatants
Source: Front Microbiol. 2022 Feb 14;12:821230. doi: 10.3389/fmicb.2021.821230 (PMC8882868; doi:10.3389/fmicb.2021.821230)
Supplement: Supplementary file 1 [file Data_Sheet_1.docx]

Supplementary Material

**Food-grade expression of manganese peroxidases in recombinant *Kluyveromyces lactis* and degradation of aflatoxin B_1_ using fermentation supernatants**

Yu Xia^1,2*^, Rui He^2^, YinSun^2^, Hangyu Zhou^3^,Minjie Gao^4^, Xiuyu Hu^5^,

Xiaobing Cui^6^, Qianqian Cheng^2^, Zhouping Wang^1,2^

^1^ State Key Laboratory of Food Science and Technology, Jiangnan University, Wuxi 214122, China

^2^ School of Food Science and Technology, Jiangnan University, Wuxi 214122, China

^3^ State Key Laboratory of Biocatalysis and Enzyme Engineering, School of Life Sciences, Hubei University, Wuhan 430062, China.

^4^ Key Laboratory of Carbohydrate Chemistry and Biotechnology, Ministry of Education, Jiangnan University, Wuxi 214122, China

^5^ China Biotech Fermentation Industry Association, Beijing 100 833, China

^6^ Anhui Heiwa food-Jiangnan University Joint R & D Center, Anhui Heiwa Food Technology Co. LTD, Bozhou 233600, China

*Corresponding author:

Yu Xia, email: [yuxia@jiangnan.edu.cn](mailto:yuxia@jiangnan.edu.cn)

**Tables**

**Table S1.**PCR verification reaction system and programs

| **PCR reaction systems** | |  | **PCR reaction programs** | |
| --- | --- | --- | --- | --- |
| system components | content |  | temperature | time |
| _dd_H_2_O | 39.5 μL |  | Step1: 95°C | 5 min |
| 10×PCR Buffer | 5.0 μL |  | Step2: 95°C | 45 s |
| dNTPs(5.0 mmol/L) | 2.0 μL |  | Step3: 55°C | 45 s |
| upstream primer(20.0 μmol/L) | 1.0 μL |  | Step4: 72°C | 45 s |
| downstream primer(20.0 μmol/L) | 1.0 μL |  | Step5: 72°C | 10 min |
| template | 1.0 μL |  | The Step2 to Step4 was circulated for 30 rounds. | |
| Taq Plus DNA Polymerase | 0.5 μL |  |  |  |

**TableS2.**Orthogonal test factors and levelsfor optimization of induction conditions

| **tests** | **A** | **B** | **C** | **D** | **E** | **F** | **G** |
| --- | --- | --- | --- | --- | --- | --- | --- |
|  | **Temperature (°C)** | **Time (h)** | **Rotation speed (rpm)** | **Hemin concentration(mmol/L)** | **pH** | **MnSO_4_concentration (mmol/L)** | **Galactose concentration (g/L)** |
| 1 | 28 | 84 | 180 | 0.8 | 5.5 | 0.8 | 50 |
| 2 | 30 | 96 | 200 | 1.0 | 6.0 | 1.0 | 60 |
| 3 | 32 | 108 | 220 | 1.2 | 6.5 | 1.2 | 70 |

**TableS3.** Orthogonal test factors and levels for degradation AFB_1_ reaction conditions

| **tests** | **A** | **B** | **C** | **D** | **E** | **F** | **G** |
| --- | --- | --- | --- | --- | --- | --- | --- |
|  | **Time (h)** | **Temperature (°C)** | **pH** | **MnSO_4_concentration (mmol/L)** | **Protein concentration(g/L)** | **Glucose concentration (mmol/L)** | **Glucose oxidase(U/mL)** |
| 1 | 32 | 35 | 4.2 | 0.8 | 3.0 | 2.2 | 1.0 |
| 2 | 36 | 40 | 4.5 | 1.0 | 4.0 | 2.5 | 1.2 |
| 3 | 40 | 45 | 4.8 | 1.2 | 5.0 | 2.8 | 1.5 |

**Table S4.**Orthogonal test results analysis of induced expression condition

| **Experiment number** | **A** | **B** | **C** | **D** | **E** | **F** | **G** | **Degradation ratio (%)** |
| --- | --- | --- | --- | --- | --- | --- | --- | --- |
|  | **Temperature (°C)** | **Time (h)** | **Speed(rpm)** | **Hemin (mmol/L)** | **pH** | **MnSO_4_ (mmol/L)** | **Galactose (g/L)** |  |
| 1 | 28 | 84 | 180 | 0.8 | 5.5 | 0.8 | 50.0 | 32.08 |
| 2 | 28 | 96 | 200 | 1.0 | 6.0 | 1.0 | 60.0 | 54.86 |
| 3 | 28 | 108 | 220 | 1.2 | 6.5 | 1.2 | 70.0 | 39.57 |
| 4 | 30 | 84 | 180 | 1.0 | 6.0 | 1.2 | 70.0 | 65.68 |
| 5 | 30 | 96 | 200 | 1.2 | 6.5 | 0.8 | 50.0 | 66.76 |
| 6 | 30 | 108 | 220 | 0.8 | 5.5 | 1.0 | 60.0 | 49.80 |
| 7 | 32 | 84 | 200 | 0.8 | 6.5 | 1.2 | 70.0 | 25.09 |
| 8 | 32 | 96 | 220 | 1.0 | 5.5 | 1.0 | 60.0 | 30.06 |
| 9 | 32 | 108 | 180 | 1.2 | 6.0 | 0.8 | 50.0 | 22.32 |
| 10 | 28 | 84 | 220 | 1.2 | 6.0 | 1.0 | 60.0 | 45.94 |
| 11 | 28 | 96 | 180 | 0.8 | 6.5 | 0.8 | 50.0 | 39.07 |
| 12 | 28 | 108 | 200 | 1.0 | 5.5 | 1.2 | 70.0 | 33.70 |
| 13 | 30 | 84 | 200 | 1.2 | 5.5 | 0.8 | 50.0 | 67.11 |
| 14 | 30 | 96 | 220 | 0.8 | 6.0 | 1.2 | 70.0 | 51.59 |
| 15 | 30 | 108 | 180 | 1.0 | 6.5 | 1.0 | 60.0 | 63.85 |
| 16 | 32 | 84 | 220 | 1.0 | 6.5 | 0.8 | 50.0 | 29.59 |
| 17 | 32 | 96 | 200 | 1.2 | 5.5 | 1.2 | 70.0 | 31.74 |
| 18 | 32 | 108 | 180 | 0.8 | 6.0 | 1.0 | 60.0 | 23.49 |
| k1 | 40.87 | 45.13 | 43.29 | 36.85 | 41.13 | 39.67 | 44.36 |  |
| k2 | 62.35 | 46.01 | 45.88 | 47.12 | 44.48 | 45.55 | 44.17 |  |
| k3 | 27.05 | 39.12 | 41.09 | 46.29 | 44.66 | 45.05 | 41.73 |  |
| Range R | 35.3 | 6.89 | 4.79 | 10.27 | 3.53 | 5.88 | 2.63 |  |
| Factor priority | ADBFCEG | | | | | | | |
| optimum proposal | A_2_D_2_B_2_F_2_C_2_E_3_G_1_ | | | | | | | |

**Table S5.**Orthogonal test results analysis of reaction condition

| **Experiment number** | **A** | | **B** | **C** | **D** | **E** | **F** | **G** | **Degradation ratio (%)** |
| --- | --- | --- | --- | --- | --- | --- | --- | --- | --- |
|  | **Time (h)** | **Temperature (°C)** | | **pH** | **MnSO_4_ concentration (mmol/L)** | **Protein concentration (g/L)** | **Glucose concentration (mmol/L)** | **Glucose oxidase (U/mL)** |  |
| 1 | 32 | | 35 | 4.2 | 0.8 | 3.0 | 2.2 | 1.0 | 37.71 |
| 2 | 32 | | 40 | 4.5 | 1.0 | 4.0 | 2.5 | 1.2 | 70.03 |
| 3 | 32 | | 45 | 4.8 | 1.2 | 5.0 | 2.8 | 1.5 | 27.84 |
| 4 | 36 | | 35 | 4.2 | 1.0 | 4.0 | 2.8 | 1.5 | 38.64 |
| 5 | 36 | | 40 | 4.5 | 1.2 | 5.0 | 2.2 | 1.0 | 73.55 |
| 6 | 36 | | 45 | 4.8 | 0.8 | 3.0 | 2.5 | 1.2 | 26.42 |
| 7 | 40 | | 35 | 4.5 | 0.8 | 5.0 | 2.5 | 1.5 | 64.51 |
| 8 | 40 | | 40 | 4.8 | 1.0 | 3.0 | 2.8 | 1.0 | 37.62 |
| 9 | 40 | | 45 | 4.2 | 1.2 | 4.0 | 2.2 | 1.2 | 56.83 |
| 10 | 32 | | 35 | 4.8 | 1.2 | 4.0 | 2.5 | 1.0 | 20.94 |
| 11 | 32 | | 40 | 4.2 | 0.8 | 5.0 | 2.8 | 1.2 | 48.72 |
| 12 | 32 | | 45 | 4.5 | 1.0 | 3.0 | 2.2 | 1.5 | 61.92 |
| 13 | 36 | | 35 | 4.5 | 1.2 | 3.0 | 2.8 | 1.2 | 68.71 |
| 14 | 36 | | 40 | 4.8 | 0.8 | 4.0 | 2.2 | 1.5 | 36.20 |
| 15 | 36 | | 45 | 4.2 | 1.0 | 5.0 | 2.5 | 1.0 | 60.94 |
| 16 | 40 | | 35 | 4.8 | 1.0 | 5.0 | 2.2 | 1.2 | 28.33 |
| 17 | 40 | | 40 | 4.5 | 1.2 | 3.0 | 2.5 | 1.5 | 75.63 |
| 18 | 40 | | 45 | 4.2 | 0.8 | 4.0 | 2.8 | 1.0 | 49.99 |
| k_1_ | 44.53 | | 43.14 | 48.72 | 43.93 | 51.35 | 49.09 | 49.09 |  |
| k_2_ | 50.74 | | 56.97 | 69.07 | 49.58 | 45.44 | 53.10 | 53.10 |  |
| k_3_ | 52.17 | | 47.32 | 29.56 | 53.93 | 50.65 | 45.25 | 45.25 |  |
| Range R | 7.63 | | 13.83 | 39.51 | 10 | 5.91 | 7.85 | 4.01 |  |
| Factor priority | CBDFAEG | | | | | | | | |
| Optimum proposal | C_2_B_2_D_3_F_2_A_3_E_1_G_3_ | | | | | | | | |

**Figures**

（A）

（B）


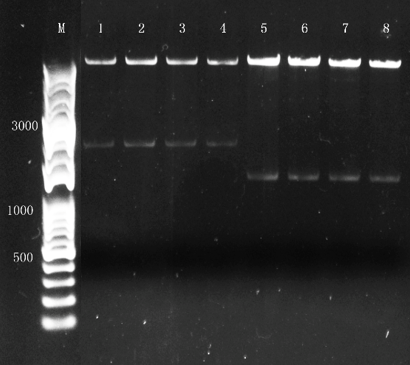

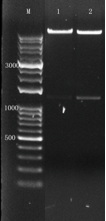


M 1 2 3 4 5 6 7 8 M 1 2

3000

1000

500

3000

1000

500

**Figure S1.**Electrophoresis of double digestion verification of recombinant plasmids pKLAC1-Phc*mnp*, pKLAC1-Phs*mnp* and pKLAC1-Plo*mnp*plasmids extracted from the DH5α transformants. (A) Double digestion electrophoresis of recombinant pKLAC1-Phc*mnp*and pKLAC1-Phs*mnp* plasmids, lane M: DNA Marker; lanes 1~4: *Bgl*Ⅱ-*Sal*Ⅰ double digestion of pKLAC1-Phc*mnp*; lanes 5~8: *Bgl*Ⅱ-*Sal*Ⅰ double digested plasmids of pKLAC1-Phs*mnp* (B) Double digested electrophoresis of recombinant DH5α(pKLAC1-Plo*mnp*) plasmids, lane M: DNA Marker; lanes 1, 2: *Bgl*Ⅱ-*Sal*Ⅰ double digested pKLAC1-Plo*mnp.*

（A）

（B）


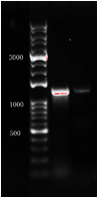

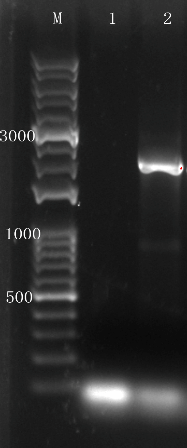


M 1 2 M 1 2

3000

1000

500

3000

1000

500

**Figure S2.**Electrophoresis ofPCR verificationresults fromthe recombinants GG799(pKLAC1-Phc*mnp*), GG799(pKLAC1-Phs*mnp*) and GG799(pKLAC1-Plo*mnp*). (A) lane 1: GG799 host genomic PCR verificationresults; lane 2: recombinant GG799(pKLAC1-Phc*mnp*) genomic PCR verificationresults. (B) lane 1: GG799(pKLAC1-Phs*mnp*) genomic PCR verification electrophoresis; lane 2: recombinant GG799(pKLAC1-Plo*mnp*) genomic PCR validation electrophoresis. Lane M: DNA standard molecular weight.

**B**

**A**

**D**

**C**

**Figure S3.** Degradation ratio of AFB_1_ by the enzyme PhcMnp under different induction conditions. (A) the effect of temperature on the degradation ratio of AFB_1_ by PhcMnp.(B)the effect of time on the degradation ratio of AFB_1_ by PhcMnp.(C)the effect of rotation speed on the degradation ratio of AFB_1_ by PhcMnp.(D) the effect of pH on the degradation ratio of AFB_1_ by PhcMnp.

**C**

**B**

**A**

**Figure S4.** Degradation ratio of AFB_1_ by PhcMnp under different induced additives concentrations.(A) the effect of hemin concentrations on the degradation ratio of AFB_1_ by PhcMnp.(B)the effect of MnSO_4_ concentrations on the degradation ratio of AFB_1_ by PhcMnp.(C)the effect of galactose concentrations on the degradation ratio of AFB_1_ by PhcMnp.
